# Mechanical Behavior of Oxide Dispersion Strengthened Steel Directly Consolidated by Rotary Swaging

**DOI:** 10.3390/ma17194831

**Published:** 2024-09-30

**Authors:** Radim Kocich, Lenka Kunčická, Petr Král, Karel Dvořák

**Affiliations:** 1 Department of Metallurgical Technologies, Faculty of Materials Science and Technology, VŠB–Technical University of Ostrava, 17. listopadu 2172/15, 708 00 Ostrava-Poruba, Czech Republic; 2 Faculty of Mechanical Engineering, Brno University of Technology, Technická 2896, 616 00 Brno, Czech Republic; 3 Institute of Physics of Materials, Czech Academy of Sciences, Žižkova 22, 616 00 Brno, Czech Republic; 4 Faculty of Civil Engineering, Brno University of Technology, Veveří 331/95, 602 00 Brno, Czech Republic

**Keywords:** rotary swaging, direct consolidation, oxide dispersion strengthening, microstructure, microhardness

## Abstract

Among the main benefits of powder-based materials is the possibility of combining different constituents to achieve enhanced properties of the fabricated bulk material. The presented study characterizes the micro- and sub-structures and related mechanical properties of ferritic steel strengthened with a fine dispersion of nano-sized Y_2_O_3_ oxide particles. Unlike the typical method of preparation via rolling, the material presented herein was fabricated by direct consolidation from a mixture of powders using the versatile method of hot rotary swaging. The mechanical properties were evaluated at room temperature and also at 1300 °C to document the suitability of the prepared steel for high-temperature applications. The results showed that the imposed shear strain, i.e., swaging ratio, is a crucial parameter influencing the microstructure and, thus, material behavior. The workpiece subjected to the swaging ratio of 1.4 already exhibited a sufficiently consolidated structure with ultra-fine grains and featured high room-temperature microhardness values (up to 690 HV0.5), as well as a relatively high maximum flow stress (~88 MPa) when deformed at the temperature of 1300 °C with the strain rate of 0.5 s^−1^. However, the dispersion of oxides within this sample exhibited local inhomogeneities. Increasing the swaging ratio to 2.5 substantially contributed to the homogenization of the distribution of the Y_2_O_3_ oxide particles, which resulted in increased homogeneity of mechanical properties (lower deviations from the average values), but their lower absolute values due to the occurrence of nucleating nano-sized recrystallized grains.

## 1. Introduction

The properties of metallic materials are primarily influenced by chemical compositions and microstructures, the latter of which is typically related to the selected deformation (thermomechanical) processing procedure and possibly applied heat treatments [1,2,3,4]. The microstructures, i.e., grain size and morphology, as well as the presence of secondary phases and precipitates, affect the behavior of the prepared materials non-negligibly [5,6]. Therefore, by combining different approaches, a wide range of metallic materials featuring various microstructures and combinations of mechanical, physical, and utility properties can be fabricated [7,8].

The majority of commonly used steels use carbides, nitrides, and possibly carbonitrides as strengthening particles [9,10]. Steels strengthened with dispersions of fine (nano-sized) oxide particles (oxide-dispersion-strengthened, ODS) were developed in the sixties of the twentieth century, primarily for the nuclear energetics industry and specific components operating under high temperatures [11,12,13,14,15]. Nevertheless, due to their excellent mechanical properties and high durability, they are also suitable for components and parts operating at ambient temperatures [16,17]. Contrary to, for example, Ni-based superalloys, the mechanical properties of which decrease significantly when operating at temperatures higher than ~800 °C [18,19,20], ODS steels are stable up to the temperatures of ~1300 °C due to the presence of nano-sized oxide particles, which effectively hinder the movement of dislocations [21,22].

Some of the ODS materials have become popular and known under their commercial labels, for example, EUROFER97 [23], a ferritic/martensitic ODS steel suitable for applications within fusion reactors [24]; MA956 [25], an iron-chromium-aluminum Incoloy ODS alloy primarily developed for aerospace applications [26]; MA957 [27], a ferritic ODS steel intended especially for liquid-metal fast breeder reactors [28]; PM 2000 [29], a Fe-20Cr-5Al ODS steel suitable for components in tubing systems within combined cycle gas turbine heat exchangers in biomass power plants [29]; or PM1000 [30], a nickel-based ODS superalloy with applications in the aerospace and glass-processing industry [31]. ODS alloys feature favorable mechanical properties, especially hardness and strength, as well as specific properties, such as high resistance against radiation [32,33]. On the other hand, their plastic properties are rather low, although some of the novel ODS alloys are able to maintain relatively high ductility, together with exceptional strength [34,35].

Among the chemical composition, an important factor affecting the behavior of ODS materials is the method of preparation, and especially the way of consolidation. To acquire bulk fine-grained materials featuring advantageous properties, ODS steels are typically fabricated by means of powder metallurgy, i.e., by mechanical alloying (MA) [36,37] or milling [38], combined with subsequent direct consolidation by compacting methods (e.g., hot isostatic pressing, HIP [39,40]), or via deformation methods, such as extrusion [41,42], rolling [43,44], forging [45,46], or their combinations [47,48].

Rotary swaging (RS) is a method of intensive plastic deformation, which is also advantageously industrially applicable, typically in the automotive. The principle of the method lies in the gradual application of increments of high-shear strain into the processed workpiece [49,50,51]. In the case of bulk materials, these increments primarily contribute to the achievement of (severe) grain refinement, while in the case of powder-based materials, they primarily promote shear mixing and homogeneous consolidation of the powders. RS features a pre-dominantly compressive stress state, which supports consolidation and grain refinement, as well as homogenization of residual stress [52]. Contrary to the methods of severe plastic deformation (SPD), i.e., methods like Equal Channel Angular Pressing (ECAP) and related techniques [53,54,55], which are also based on imposing (increments of) high shear strain into the processed materials [56], RS also imparts changes in the shapes and dimensions of the workpieces and thus enables to achieve both solid and hollow components of various geometries [57]. Moreover, the length (i.e., volume) of the processed workpieces is theoretically unlimited [58]. RS is thus so versatile that it can be used to fabricate components with virtually any required dimensions from virtually any material, regardless of its formability [59,60,61]. The method is suitable for preparing products and components with not only circular but also complex cross-sections, all with surfaces of exceptional quality. Last but not least, RS can advantageously be used to consolidate ODS powders directly.

The presented study deals with the direct consolidation of ferritic ODS steel powder using RS under hot conditions. The canned powders were subjected to various swaging ratios, and the consolidated samples were subjected to detailed observations of micro- and sub-structures, as well as to assessment of the mechanical behavior at room temperature (Vickers microhardness testing) and at the temperature of 1300 °C (compression testing).

## 2. Materials and Methods

### 2.1. Experimental Material

**Figure 1:**
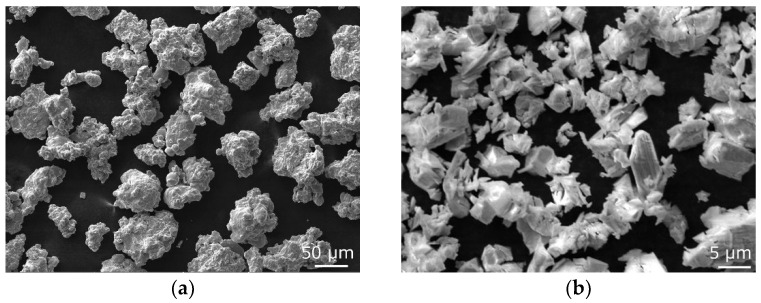
SEM secondary electron images of original powders: Fe (**a**); Y_2_O_3_ (**b**).

The first processing step involved the preparation of the powder mixture to be subsequently directly consolidated. Fe powder with additions of Al (10 wt.%), Cr (4 wt.%), and Y_2_O_3_ (3 wt.%) was prepared with the use of mechanical alloying (MA) in an evacuated attritor of own construction filled with bearing balls and rotating at 70 RPM. MA not only generally ensures sufficient mixing of the original powders but also provides gradual fragmentation of the particles, which contributes to mixture homogenization and decreases the grain size [62]. Additions of Al and Cr were selected to increase the oxidation resistance of the consolidated material and thus prospectively increase its lifetime, while the addition of Y_2_O_3_ was selected to provide the consolidated material with exceptional mechanical properties and resistance at elevated temperatures by the effect of grain boundary pinning. Figure 1a shows a scanning electron microscopy (SEM-SE) image of the original Fe powder, while Figure 1b shows a SEM-SE image of the Y_2_O_3_ powder.

After the MA, the powder mixture was filled into steel containers with 50 mm in diameter and vacuum sealed. The inside walls of the containers were covered with Al_2_O_3_ powder to prevent the container wall from sticking to the final consolidated material and facilitate stripping. The sealed containers were then subjected to RS at 900 °C for a direct consolidation of the powder mixture. Induction heating was applied to ensure homogeneous temperature distribution throughout the container during the entire swaging pass. The swaging was performed with a total swaging ratio of 2.5; however, samples were already acquired at a swaging ratio of 1.4 to assess the progress of structure development during the consolidation. The swaging ratio of 1.4 was selected based on our previous preliminary study on the feasibility of application of the RS process for hot consolidation of such powders, during which this particular swaging ratio was shown to be minimum for successful consolidation of the powders [63]. The mentioned study also proved that continuing swaging results in further substructure development, resulting in related changes in the mechanical properties. In other words, the applied swaging ratio directly influences the final behavior of the consolidated material. The primary hypothesis was thus to further test the material from the viewpoint of its technological limits. Therefore, the consolidated rod was subjected to RS with the highest swaging ratio the material could withstand without exhibiting cracking and failure; this was shown to be the value of 2.5. The swaging ratio was calculated using Equation (1),

(1) $$\varphi =\ln\left(\frac{S_{0}}{S_{n}}\right)$$

where *S*_0_ and *S_n_* are cross-sectional areas of container at input and output to swaging dies, respectively.

### 2.2. Analyses

The distributions of Y_2_O_3_ oxide particles within the consolidated microstructures were assessed by scanning electron microscopy—secondary electrons observations (SEM-SE). Given the nano-sized grains and extremely high accumulation of lattice defects, microscopic observations of the individual grains on conventional bulk samples could not be performed with sufficient reliability. For this reason, all the structure analyses (except secondary electron observations of the homogeneity of distribution of the oxides) were performed on TEM (transmission electron microscopy) foils. The foils were prepared by manual grinding and final electrolytic polishing. The analyses were primarily performed using the JEM-2100 transmission electron microscope (TEM) (JEOL, Tokyo, Japan) operating at 200 kV. In order to observe (relatively) larger areas of the microstructure, additional analyses on the foils via SEM—EBSD (electron backscatter diffraction) (Tescan Lyra 3 XMU FEG/SEMxFIB equipment with Symmetry EBSD detector, Tescan Orsay Holding a.s., Brno, Czech Republic) were performed. The scanning was performed on a TEM foil with the scan step of 20 nm in order to reliably document the nano-sized structural features. The analyses were evaluated using the AZtec Crystal 3.1 software (Oxford Instruments Nanotechnology Tools Limited, Abingdon, UK). For the EBSD evaluations, the considered limiting values were 5° and 15° for LAGB and HAGB (low-angle grain boundaries high angle grain boundaries, respectively).

The mechanical properties of the consolidated material were assessed by microhardness testing at room temperature and supplemented with compression tests at 1300 °C at various strain rates. The investigations of Vickers microhardness were performed with a load of 1 kg and load time of 10 s for each indent using a FM ARS 900 device (Future-Tech Corp., Kawasaki, Japan). The measurements were performed across the cross-sections of the perpendicularly cut consolidated samples, with a spacing of 0.5 mm. The compression tests were performed with the use of a Gleeble 3800 universal thermal-mechanical physical simulation machine equipped with a Hydrawedge mobile conversion testing unit (all Dynamic Systems Inc., Poestenkill, NY, USA). The Hydrawedge unit is purposefully designed for hot compression testing in a wide range of temperatures and strain rates. Samples from both the consolidated rods, i.e., swaging ratios of 1.4 and 2.5, were tested in the form of cylindrical compression test samples with a diameter of 10 mm and length of 15 mm. The testing was performed at the temperature of 1300 °C in combination with the strain rates of 0.005 s^−1^, 0.05 s^−1^, and 0.5 s^−1^. Each testing sample was heated directly to the deformation temperature with the heating rate of 10 °C·s^−1^ (via direct electric resistance heating), followed by a time dwell of 5 min to homogenize the temperature throughout the sample. The temperature measurement was performed by a pair of thermocouple wires of R-type (Pt-13%Rh (+), Pt (−)) welded on the surface of the sample in the middle length. Graphite foils and nickel-based grease were utilized to protect the anvils and reduce friction forces on the anvils–sample interface. During the testing, the testing chamber was held under a high vacuum (below 10^−2^ Torr) to hinder possible oxidation processes.

## 3. Results

### 3.1. Microstructure Homogeneity

**Figure 2:**
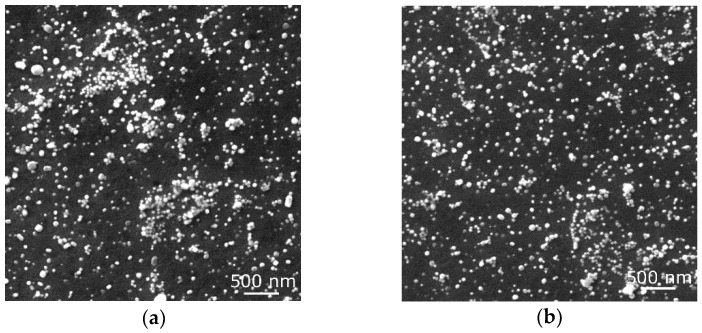
SEM-SE scans of microstructures consolidated with swaging ratios: 1.4 (**a**); 2.5 (**b**).

Firstly, SEM-SE observations of the consolidated materials were performed. Figure 2a shows an image of the microstructure of the workpiece subjected to the swaging ratio of 1.4. As can be seen, the microstructure was already sufficiently consolidated and did not exhibit any presence of macroscopic voids. However, its homogeneity was not satisfactory. In other words, the microstructure of the material consolidated with the swaging ratio of 1.4 exhibited the presence of local clusters of powder particles, as seen in Figure 2a. Increasing the swaging ratio to 2.5 then evidently contributed to the homogenization of the distribution of the oxide particles, as documented by Figure 2b. The microstructure was fully consolidated with no residual porosity and featured a more or less homogeneous distribution of the oxide particles.

### 3.2. Microstructure Assessment

The analyses involved observations of the microstructures and individual grains by acquiring EBSD data on the TEM foils prepared from cross-sectional cuts of the workpieces consolidated with both the swaging ratios.

**Figure 3:**
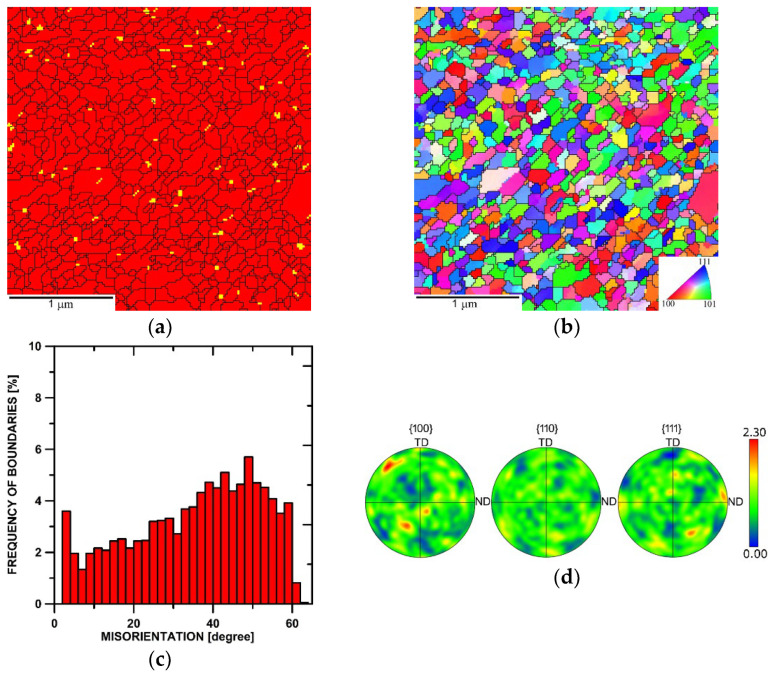
Microstructure data for the structure of the sample consolidated with a swaging ratio of 1.4: phase map (**a**); OIM (**b**); boundaries misorientations (**c**); PF texture images (**d**).

Figure 3a shows the phase map of the microstructure of the sample consolidated with a ratio of 1.4. Although the Y_2_O_3_ oxide particles were distributed more or less homogeneously throughout the microstructure, they tended to form clusters (as also documented in Figure 2a) at the grain boundaries. The OIM (Orientation Image Map) image depicting the individual grains within the respective consolidated microstructure is then in Figure 3b. The image shows that the microstructure was already consolidated and featured the majority of fully developed grains, the average grain size was 120 nm (measured via equivalent diameter), and the HAGB fraction was 87% (see the grain boundaries misorientation chart in Figure 3c). Figure 3d then shows the pole figure (PF) texture plots depicting the orientations of the grains within the sample. According to the PFs, the maximum texture intensity was about two times random, which points to the fact that no significant texture developed within the consolidated microstructure swaged with a ratio of 1.4 (as also noticeable from the OIM image in Figure 3b).

**Figure 4:**
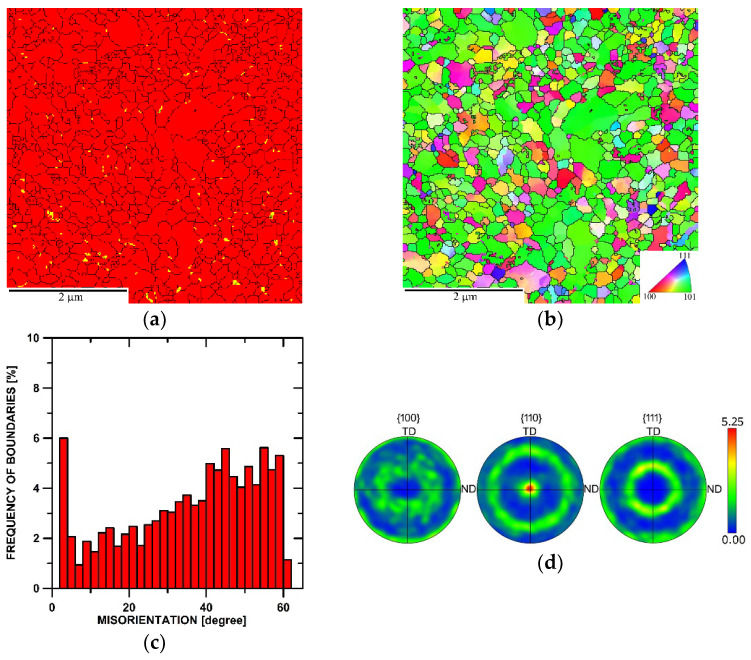
Microstructure data for the structure of the sample consolidated with a swaging ratio of 2.5: phase map (**a**); OIM (**b**); boundaries misorientations (**c**); PF texture images (**d**).

Figure 4a depicts the phase map of the microstructure of the sample consolidated with a swaging ratio of 2.5. As can be seen, the distribution of the Y_2_O_3_ oxide particles was more homogeneous when compared to the 1.4 ratio sample, and the particles were primarily present at the boundaries of the grains (although many of the nano-sized oxide particles were most probably below the resolution level of EBDS). This finding is further confirmed by the TEM data presented in Section 3.3. The corresponding OIM image showing the distribution of the individual grains within the microstructure is depicted in Figure 4b. The swaging ratio of 2.5 evidently ensured sufficient consolidation, and the powders transformed into a more or less homogeneous bulk structure with no evident presence of pores. The average grain size for the examined sample was 170 nm (equivalent diameter), and the majority (85%) of the grains featured HAGB, as documented by the grain boundaries misorientation chart in Figure 4c. Figure 4d then shows the orientations of the grains via PF plots and reveals that the structure featured <110>||SD fiber texture, i.e., the <110> directions were parallel with the swaging direction (this finding is supported by the OIM coloring in Figure 4b).

### 3.3. Substructure Development

**Figure 5:**
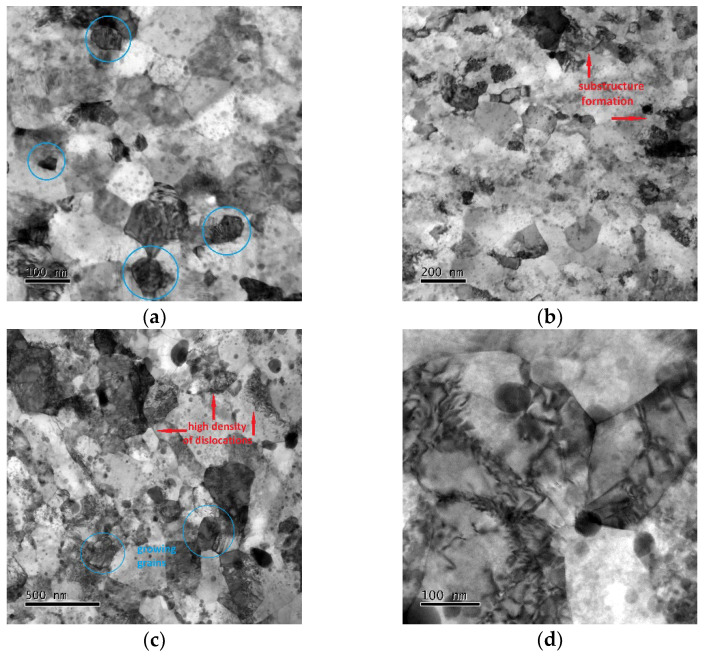
TEM images of substructure consolidated with swaging ratios: 1.4 (**a**,**b**); 2.5 (**c**,**d**). In the figures, blue circles depict growing and newly developed grains, while red arrows point to locations with developing substructure (high density of dislocations).

The level of substructure development within both the examined material states was further assessed by TEM investigations. Figure 5a,b show bright field (BF) TEM images of the substructure of the sample subjected to the ratio of 1.4. Evidently, the structure was sufficiently consolidated and featured ultra-fine (UF) and even nano-sized grains (examples of some well-developed grains are marked in blue circles in Figure 5a for better clarity). Nevertheless, inhomogeneities regarding substructure development could be seen in numerous locations of the sample. In other words, grains featuring HAGBs were not fully developed throughout the entire consolidated structure, as the substructure to be developed into full grains could be noticed. See Figure 5b to observe examples of locations featuring substructure formation. The substructure of the sample subjected to the swaging ratio of 2.5 can then be seen in Figure 5c,d. This sample featured a developed consolidated structure. However, the supposedly original grains were, at numerous locations, present together with newly recrystallized finer grains occurring at their boundaries; see Figure 5c for a better observation of the characterized structural features. Some of the grains also featured developed substructures and high dislocation density (see Figure 5d).

As regards the distribution of the oxide particles, the TEM images confirmed that the oxide dispersion was more homogeneous within the sample consolidated with the swaging ratio of 2.5. Figure 5d documents that the oxide particles were primarily present at the boundaries of the (newly recrystallized) grains and that they also effectively acted as obstacles providing dislocations pinning.

### 3.4. Mechanical Properties at Room Temperature

**Figure 6:**
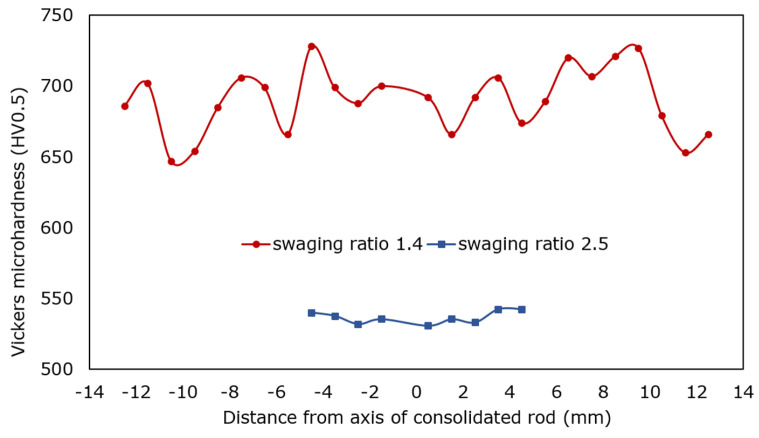
HV0.5 Vickers microhardness for consolidated samples.

Room-temperature mechanical properties of the consolidated ODS steel samples were assessed by HV0.5 Vickers microhardness measurements. The results of the measurements performed across the cross-sections of the acquired samples are summarized in Figure 6 (note that the diameters of the individual consolidated samples differ according to the applied swaging ratio, i.e., imposed total strain). The Figure shows that the sample consolidated with the swaging ratio of 1.4 featured very high microhardness values; the average microhardness for this sample was 690.1 HV0.5. However, the standard deviation for this sample was also high (22.4). Increasing the swaging ratio to 2.5 resulted in a decrease in the overall microhardness; the average value for this sample was 536.7 HV0.5. Nevertheless, the standard deviation from the average value decreased remarkably to 4.2 for this sample.

### 3.5. Mechanical Properties at Elevated Temperature

**Figure 7:**
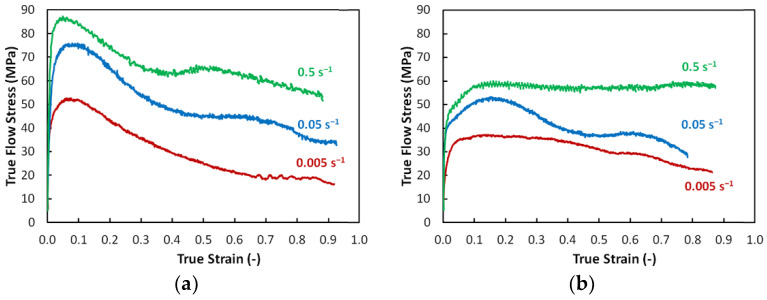
Hot flow stress response of samples consolidated with swaging ratios: 1.4 (**a**); 2.5 (**b**).

The performed hot compression tests provided experimental data on hot flow stress behaviors for both workpieces. The results of testing of samples from the workpiece consolidated with the swaging ratio of 1.4 are depicted in Figure 7a, while the resulting flow stress curves acquired from testing of samples taken from the workpiece consolidated with the swaging ratio of 2.5 are depicted in Figure 7b. As can be seen, the flow stress responses of both the workpieces to the external load were different. The samples consolidated with the swaging ratio of 1.4 exhibited generally higher stress values. Also, contrary to the flow stress responses of the samples consolidated with the swaging ratio of 2.5 exhibiting more or less steady state-like behavior, all the flow stress curves acquired for the samples subjected to the ratio of 1.4 exhibited an increase to the maximum value at the beginning of loading (at true strain of approximately 0.06), followed by a more or less gradual decrease. Comparing the respective true flow stress curves acquired at identical strain rates for both the examined material states, they met at true strain values approximately between 0.7 and 0.8 and then further decreased—the decrease was more significant for samples consolidated with the swaging ratio of 1.4, as the respective curves more or less maintained their continuing trends further on.

## 4. Discussions

The primary aim of this research was to assess the effects of the imposed swaging ratio on the effectivity of consolidation of an oxide dispersion-strengthened steel-based powder mixture via hot rotary swaging. When examining the consolidated microstructures, the differences imparted by different swaging ratios were evident. As documented by the acquired results, great differences could be seen not only in the distribution of the oxide particles within the consolidated microstructures but also in the mechanical behaviors of the consolidated workpieces. Increasing the swaging ratio evidently increased the homogeneity of distribution of the oxide particles. Within the final consolidated material (swaging ratio of 2.5), the Y_2_O_3_ oxide particles were more or less homogeneously distributed and occurred primarily at the boundaries of the grains, which subsequently favorably affected the mechanical behavior of the ODS steel by providing the grain boundary pinning effect [64,65].

Considering the homogeneous dispersion of the nano-sized Y_2_O_3_ particles, the consolidated material can be characterized as a nanocomposite. Microstructure and substructure observations also documented that the behavior of the material consolidated with the swaging ratio of 2.5 was comparable to that of conventionally prepared (cast) materials. Within the final workpiece, the original powders were well consolidated, and the microstructure developed via the chain of dislocations generation and movement, formation of dislocations cells and walls, formation of subgrains, and subsequent development of full refined grains (as seen, e.g., in Figure 5c,d) [66]. The effect of additions of oxide particles was primarily in hindering the movement of dislocations and also boundaries of the (newly emerging, i.e., recrystallized) grains [67]. The first mentioned phenomenon significantly contributed to the high microhardness at room temperature and favorable mechanical properties at very high temperatures, whereas the latter primarily contributed to grain refinement and conservation of nano-sized grains—which, advantageously, contributed to increasing the mechanical properties of the consolidated ODS steel, too.

Together with the very fine grain size, the addition of oxide particles provided the consolidated material with exceptional room-temperature mechanical properties; the microhardness reached almost 700 HV0.5 for the sample consolidated with a ratio of 1.4. Nevertheless, this sample also featured a relatively high standard deviation from the average microhardness value. This can primarily be attributed to the above-mentioned inhomogeneous distribution of the oxide particles within the material consolidated with the lower swaging ratio, as well as the possible presence of local inhomogeneities (i.e., local microscopic voids could still be present). Increasing the swaging ratio then resulted in a decrease in the standard deviation, as the microstructure featured greater homogeneity. Nevertheless, the average microhardness value for the sample consolidated with the swaging ratio of 2.5 decreased substantially.

A similar trend in the mechanical behavior of the consolidated workpieces was observed also at the elevated temperature of 1300 °C. The samples swaged with the ratio of 1.4 exhibited generally higher flow stress values, although the flow stress curves exhibited decreasing trends with increasing strain, while the samples swaged with the ratio of 2.5 featured generally lower flow stress values. On the other hand, the stability of flow stress with increasing strain was greater for the 2.5 swaging ratio samples compared to the 1.4 swaging ratio ones, i.e., the flow stress curves exhibited steady state-like behavior (Figure 7b). The differences in the flow stress response of both the consolidated pieces can be attributed to different amounts of the imposed shear strain, which are directly related to the swaging ratios. In other words, increasing the swaging ratio goes hand in hand with increasing the imposed strain, related differences in microstructure development imparted by differences in the interactions between the hardening/softening processes, and finally, also influencing the stress level and distribution within a swaged material [68,69,70]. The flow stress responses further imply that both the consolidated steels exhibited dynamic softening processes during the high-temperature testing. However, the type and extent of the occurring softening processes differed according to the imposed swaging ratio. In other words, the lower imposed strain resulted in a generally higher flow stress and imparted a more significant softening than the higher imposed strain. This behavior is most probably related to the level of energy accumulated within the microstructures during the ODS steel processing [71]. Firstly, the powder mixture was prepared by MA, during which some energy was already imposed and stored within the powder particles, which should be considered. The major portion of energy was then accumulated within the consolidated material during the hot swaging. The fact that during hot rotary swaging, the chain of work hardening-restoration repeatedly occurs is generally known; moreover, the consolidated workpieces were subjected to the influence of the hot temperature for different time periods (according to the applied swaging ratios). Therefore, the time provided for the mentioned hardening-restoring chain to repeat was shorter for the workpiece consolidated with the swaging ratio of 1.4; this primarily influenced the restoring part, as softening processes could not fully develop. On the other hand, both the dynamic/static softening processes could develop to a greater extent within the workpiece consolidated with the swaging ratio of 2.5.

The subsequently performed hot compression tests confirmed these differences, which were revealed as generally lower flow stress values and also the dominant presence of dynamic recovery instead of recrystallization, particularly for the highest strain rate for the sample consolidated with the swaging ratio of 2.5. The sample consolidated with the swaging ratio of 1.4, on the contrary, clearly exhibited dynamic recrystallization, especially at the higher strain rates, the flow stress curves for which featured multiple peaks confirming the interaction of hardening-restoring. Let us consider, for example, the flow stress curve acquired at the strain rate of 0.5 s^−1^ (Figure 7a). The early peak point occurrence was followed by a significant flow stress decrease and subsequent transition to a steady state. However, the steady-state flow was locally interrupted by another, though less remarkable, flow stress decrease; such behavior can usually be observed during the deformation of metallic materials at relatively high temperatures and low strain rates. This mechanical behavior corresponded with the TEM observations; Figure 3a shows that the microstructure featured fine consolidated grains and local inhomogeneities regarding structure development, and with continuing deformation, the energy accumulation–recrystallization chain occurred. During testing of the sample swaged with the ratio of 2.5, the dynamic recrystallization was less pronounced, and the corresponding flow stress curve implied the occurrence of dynamic recovery, as the curve exhibited more or less steady state-like behavior (Figure 7b).

## 5. Conclusions

A mechanically alloyed mixture of steel-based powders with the addition of nano-sized Y_2_O_3_ oxide particles was directly consolidated using rotary swaging under hot conditions, and the effects of the applied swaging ratios were assessed. The basic acquired conclusions are the following:

- swaging ratio of 1.4 resulted in a consolidated microstructure featuring ultra-fine grains and advantageous mechanical properties (microhardness of 690 HV0.5), but the consolidated workpiece still featured local inhomogeneities in Y_2_O_3_ particles distribution;
- swaging ratio of 2.5 substantially contributed to the homogenization of Y_2_O_3_ particle distribution and thus homogeneity of the mechanical properties. Mechanical properties exhibited lower absolute values given by occurring recrystallization and a lower extent of accumulation of lattice defects;
- testing at 1300 °C and multiple strain rates revealed generally higher absolute flow stress values, but flow stress decreased after reaching peak strain for the workpiece with a swaging ratio of 1.4 (development of dynamic recrystallization), and lower flow stress values but steady state-like behavior for workpiece with a swaging ratio of 2.5 (development of dynamic recovery)

The study clearly documents that rotary swaging is highly suitable for direct consolidation of ODS powder-based steels and that their behavior can be tailored by optimizing the swaging conditions, e.g., via modifying the swaging ratio. The ongoing following research aims to further investigate the durability of the prepared materials at high temperatures, possibly by long-term creep testing, in order to confirm the applicability of the consolidated material, e.g., for durable components of machines operating at high temperatures (e.g., creep testing machines).

## Data Availability

The original data supporting the research is not publicly available, but a portion of the data that is not confidential is available upon request from the corresponding author.
